# Yttria-Calcia-Co-Stabilized Tetragonal Zirconia Polycrystals Made by Powder Mixing

**DOI:** 10.3390/ma19061205

**Published:** 2026-03-19

**Authors:** Selina Grübel, Bettina Osswald, Frank Kern

**Affiliations:** Institute for Ceramic Materials and Composites, Faculty 7: Engineering Design, Production Engineering and Automotive Engineering, University of Stuttgart, Allmandring 7b, 70569 Stuttgart, Germanybettina.osswald@ikmt.uni-stuttgart.de (B.O.)

**Keywords:** zirconia, mechanical properties, microstructure, phase composition, co-stabilization, yttria, calcia

## Abstract

**Highlights:**

**Main findings:**

- Yttria-calcia-co-stabilized zirconia can be obtained by powder blending;
- The values of mechanical properties and Low temperature degradation (LTD) resistance are between those of Y-TZP (tetragonal zirconia polycrystalline) and Ca-TZP;
- Low sintering temperature favors strength and LTD resistance.

**Implications of the main findings:**

- Y-Ca-TZP materials may be interesting for biomedical applications;
- Further improvements may be possible by adjusting fractions and stabilizer contents of the starting powders;
- Powder mixing provides easy access to some standard compositions.

**Abstract:**

In this study, 1.5Y-2.2Ca-TZP materials were obtained by hot pressing of a mixed and milled blend of 3Y-TZP and 4.4Ca-TZP powders. The materials were sintered at temperatures between 1250 °C and 1400 °C and characterized with respect to mechanical properties, microstructure, phase composition and stability against low-temperature degradation. In the tested range, the bending strength of the TZP decreases with increasing sintering temperature from 1300 MPa to 1050 MPa while the toughness shows a rising trend from 5 MPa√m to 8 MPa√m. The grain size distribution in the microstructure is broad with average grain sizes increasing from 150 nm to 250 nm with rising sintering temperature. LTD tests revealed high aging resistance for TZP sintered at 1300 °C. The Y-Ca-co-stabilized TZP equilibrates the properties of Ca-TZP and Y-TZP.

## 1. Introduction

Yttria-stabilized zirconia materials have become commodities in recent decades. They are applied in mechanical engineering and biomedical applications such as dental implants due to their high strength, fine grain size and high fracture resistance [1]. The excellent mechanical properties result from transformation toughening (TT), a stress-induced martensitic phase transformation from metastable tetragonal to stable monoclinic phase [2]. Yttria added as stabilizer forms a solid solution with zirconia. As yttrium is trivalent, oxygen vacancies are introduced for charge compensation. These vacancies reduce the coordination number of the zirconium cations and distort the zirconia lattice [3]. Hereby, the high-temperature polymorphs can be retained metastable after sintering. Yttria as an oversized dopant tends to segregate to the grain boundaries, which restrains grain growth due to solute drag. Alumina is frequently added in small amounts to slow down grain growth [4]. The most common formulation 3Y-TZP contains 3 mol% Y_2_O_3_. At industrial scale, the powders are produced by co-precipitation or hydrolysis of yttrium and zirconium salts, which leads to an initially homogeneous stabilizer distribution [5,6]. With respect to thermodynamics, 3Y-TZP is a composition in the t + c field (miscibility gap) at a typical sintering temperature of ~1400 °C [7]. Hence, under equilibrium conditions at this composition, we may expect a segregation into tetragonal solid solution containing ~2.5 mol% Y_2_O_3_ (major constituent) and cubic solid solution containing 6.5 mol% Y_2_O_3_. However, this segregation is kinetically hindered as it requires uphill diffusion. Complete segregation requires high sintering temperatures and/or long dwell [8,9]. The most prominent disadvantage of Y-TZP is the vulnerability against low-temperature degradation (LTD) [10]. In moist environments, hydroxy species such as water enter the oxygen vacancies and increase the coordination number of zirconium cations. Therefore, they de-stabilize the tetragonal phase. The degradation is slow but relevant for applications in medical implants, which are implanted into the body for decades [11]. LTD of TZP and its composites is a critical issue in biomedical applications as the sudden failure of total hip or knee endoprostheses leads to pain and sudden immobility for the patient and inevitably requires revision surgery. Hence, Y-TZP is no longer used for hip implants in Europe and the US. In the case of dental applications such as implants and restorations (crowns, bridges), a failure is less detrimental as the implants are accessible. For dental applications, the minimum requirements for LTD according to DIN EN ISO 13356:2016 is <25% monoclinic after 5 h of accelerated autoclave aging at 134 °C [12].

It is commonly believed that LTD follows a nucleation and growth process in which single surface grains transform, expand, and put the surrounding grain boundaries under stress. This favors the penetration of the fluids along the grain boundaries into the volume of the component [13]. However, Keuper et al. showed that LTD actually follows linear kinetics [14]. LTD may lead to surface roughening, breakout of lumps or detachment of whole surface layers, which may destroy the TZP components [15].

In the last few years, some important new trends in research concerning TZP materials for structural applications can be identified. The first one is the application of Y-TZP materials with reduced stabilizer contents in order to boost the toughness. Reducing the stabilizer content to values as low as 1.5–2 mol% Y_2_O_3_ results in toughness values of 8–9 MPa√m. This is a drastic improvement compared to standard 3Y-TZP. On the other hand, this shifts the strength–toughness relations into the transition range between defect size- and transformation-controlled failure [16]. Kern and Imariouane [17,18] provide a good overview on the properties of these low-yttria TZPs and the recent literature.

Composites of ceria-stabilized zirconia with alumina and hexa-aluminates combine an extremely transformable TZP matrix with dispersions of second phases which suppress the autocatalytic transformation behavior of Ce-TZP. Therefore, extremely damage-tolerant TZP-composites exploiting transformation-induced plasticity (TRIP) are obtained. These materials cannot compete with Y-TZP in terms of strength but offer high stability against LTD [19,20,21].

Recently, TZP materials combining up to five different trivalent rare earth oxide stabilizers have been elaborated [22,23]. These materials also exploit TRIP. They provide higher strength and slightly lower toughness than Ce-TZP composites. The latest trend is the revival of Ca-TZP materials. Here, the motivation is to replace yttria, which is nowadays classified as a critical raw material, by inexpensive and ubiquitous calcia.

Calcia-stabilized zirconia as Ca-PSZ, in which tetragonal grains are embedded into a cubic matrix, was the first technically applied transformation-toughened zirconia material [24]. Ca-TZP consisting entirely of tetragonal grains was first described by the group of Haberko [25,26]. It was produced by sintering ultrafine powders at low sintering temperature (~1200 °C). The material offers a slightly lower strength and a slightly higher toughness than 3Y-TZP. The most prominent issue is, however, the very low critical grain size of 100–140 nm. Above the critical grain size, spontaneous failure is observed [26,27]. Calcia, being larger than yttria, tends to segregate even more to the grain boundary and slows grain growth. This effect was used by Li et al. to limit the grain growth of Ce-TZP by co-doping with calcia [28]. For two decades, Ca-TZP was more or less a scientific curio. However, recently, a high-quality powder became available, which makes the material attractive for use in medical applications as the studies performed showed a combination of excellent mechanical properties and LTD resistance [29,30].

Yttria-calcia-co-stabilized TZP materials have been scarcely investigated. It is unclear whether one of the two constituents dominates in a mixture and how co-stabilization affects mechanical properties, microstructure evolution, possible critical grain sizes and LTD.

The first paper combining yttria and calcia as stabilizers in TZP was published last year [31]. Feng et al. obtained co-stabilized powders by co-precipitation and tried compositions stabilized with 2 and 3 mol% CaO with additions of 0.5–2 mol% of Y_2_O_3_. The powders were ultrafine; the pressureless sintered samples showed a fine microstructure and mechanical properties strongly depending on the recipes. As the samples were small, only a basic mechanical characterization (excluding bending strength) was carried out.

The co-stabilization with yttria and calcia seems attractive. Powders with BET surface areas of >100 m^2^/g, which were used in the study by Feng [31], are, however, almost unprocessable in conventional shaping processes for ceramics components. Hence, in the present study, we tried a mixing and milling method combining equal amounts of commercially available 3Y-TZP and 4.4-Ca-TZP powders (which we had previously tested separately) to obtain a powder blend for 1.5Y-2.2Ca-TZP [30,32]. The choice of this exact composition is an obvious starting point for further studies. We try to obtain a first impression, if the blending technology, which presents a simple approach, is capable of obtaining such co-stabilized TZP with attractive mechanical properties and LTD resistance. Moreover, we try to elucidate whether the blending process leads to particular changes compared to the more homogeneous co-precipitated starting materials applied by Feng et al. [31].

## 2. Materials and Methods

The starting powders used for this study are 4.4Ca-TZP (HSY-481, Daiichi Kigenso Kagaku Kogyo, Osaka, Japan; S_BET_ = 23.1 m^2^/g; stabilizer content, 4.4 mol% CaO; alumina content, 0.25 wt.%) and 3Y-TZP (Innovnano, Coimbra, Portugal; primary particle size, 50 nm; stabilizer content, 3 mol% Y_2_O_3_; alumina content, 0.4 wt.%). The 3Y-TZP is made from a mixture of yttrium and zirconium precursors by detonation synthesis [33,34]. A 300 g batch composed of equal weight fractions of both powders was attrition-milled in 500 mL 2-propanol with 3Y-TZP balls of 2 mm in diameter for 4 h at 1000 rpm. Subsequently, the milling media were separated and the suspension was dried overnight at 70 °C. The dry powder was screened with a 200 µm mesh.

The samples were consolidated by hot pressing in a boron nitride-clad graphite die at 60 MPa of axial pressure for 1 h in vacuum. The sintering temperatures were varied between 1250 °C and 1400 °C in 25 °C increments. Heating was carried out with approximately 50 K/min, and the cooling was a free cooling process by shutting the heater off and filling the compartment with argon to enable convective heat transfer.

Two disks of 40 mm in diameter and 17 g in weight separated by a graphite spacer were sintered simultaneously. This results in disks with a thickness of ~2.2 mm (0.1 mm of machining allowance per side for a target value of 2 mm after machining).

The disks were manually de-burred with a 40 µm diamond disk and lapped on both sides with a 15 µm diamond suspension until the sintering skin was removed and the disks were level. One side was polished subsequently with 15 µm, 3 µm and 1 µm diamond suspensions to a mirror-like finish. The disks were then cut into bending bars of 4 mm in width with a diamond wheel. A total of 12 bending bars were obtained per sample set. After machining, the final bending bars had sizes of 3.5 +/− 0.1 mm in width, 1.9 +/− 0.1 mm in thickness, and a length exceeding 25 mm.

The density was measured by the buoyancy method (Kern, Lörrach, Germany). Young’s modulus and Poisson’s ratio by ultrasonic excitation (IMCE, Genk, Belgium) were determined on entire disks prior to cutting. Vickers hardness HV10 (five indents, Bareiss, Oberdischingen, Germany, load 98.1 N, 10 s dwell) was measured by indentation of the polished samples. The bending strength was determined by 4 pt bending tests in a setup with a 20 mm outer and 10 mm inner span at a crosshead speed of 0.5 mm/min (Zwick-Roell, Ulm, Germany). The fracture toughness was measured by three indentation-based methods. First, by direct crack length measurement of the crack patterns of the HV10 indents (K_DCM_), the toughness was calculated according to the Palmqvist crack model of Niihara [35]. Secondly, by indentation strength in bending (K_ISB_), two bending bars were notched with four HV10 indents at a distance of 2 mm, aligned along the middle axis of the polished tensile side of the bending bar. The indented region was placed in the inner span of the above-mentioned 4 pt setup. The residual strength was determined at a crosshead speed of 2.5 mm/min immediately after indentation. The toughness was calculated using the model of Chantikul et al. [36]. The third method introduced by Braun and Lawn is an extension of the ISB protocol [37]. As only one of the four indents leads to failure, the extended crack lengths of the survivors can be employed to calculate the toughness K_LWN_. Both the LWN and ISB methods are originally based on the assumption of semicircular cracks (geometry factor ψ = 1.27). In case of tough TZP materials, the crack profiles are considerably flatter [38]; hence, a tentative correction of the geometry factor to ψ = 1.08 was applied. This value was determined for 3Y-TZP by Dransmann et al. [39]. Therefore, the ISB and LWN toughness values are reduced by a factor of 1.08/1.27. The phase composition of the polished material was determined by XRD (X’Pert MPD, Panalytical, Eindhoven, The Netherlands; CuKα1, Ge-monochromator, Bragg–Brentano setup, accelerator detector). The characteristic diffraction peaks in the 27–33° 2θ scale were integrated to quantify the tetragonal and monoclinic phase according to Toraya et al. [40]. The 72–75° 2θ range allows us to calculate the tetragonality of the zirconia from the location of the fourth-order tetragonal peak pair and identify the formation of a cubic phase. The quantification of the tetragonal and monoclinic phase in fracture surfaces allows us to determine the transformability of the zirconia. The depth h of the transformation zone [41] as well as the transformation toughness increments ∆K_IC_^T^ were calculated according to McMeeking and Evans from XRD data, Young’s modulus, and Poisson’s ratio [42].

High-resolution SEM images were taken from polished and thermally etched (1150 °C/1 min, air) surfaces. The uncorrected average grain size of the microstructure was determined by measuring the diameter of all grains in images of 100,000× magnification (>150) using imageJ software (version Java 1.8.0 internal, National Institutes of health USA). A size correction factor of 1.56 as described by Mendelson was applied in the final size distribution [43].

Low-temperature degradation was determined by an accelerated autoclave test. It is commonly assumed that 1 h in the autoclave at 134 °C corresponds to approximately 3 years in vivo. For this test, some cold pressed binderless compacts of 30 mm in diameter and 1 mm in thickness were sintered in air at 1300 °C and 1350 °C for 2 h. The two sintering temperatures were chosen in order to test materials of different grain sizes and to obtain a first impression of the influence of sintering temperature. These materials were polished and exposed to saturated water vapor in an autoclave at 134 °C for durations of 1 h, 3 h, 10 h, 30 h and 100 h. The phase composition was tested by XRD after the respective aging times. Assuming an MAJ (Mehl Avrami Johnson) nucleation and growth mechanism as proposed by Chevalier, ln(ln(1/(1 − (V_m,t_ − V_m,0_)))) was plotted vs. ln(t) with V_m,t_ as the monoclinic content at the respective time and V_m,0_ as the initial monoclinic content of the pristine sample [44]. According to MAJ, the relationship between aging time and monoclinic formation can be expressed as f = 1 − exp [(−bt)^n^], with f = V_m,t_ − V_m,0_ as the monoclinic content (in our case, V_m,0_ > 0). The rate constant b is temperature-dependent and n is the nucleation factor. The nucleation factor n can be obtained as the slope of the above-mentioned plot. ln(b) is the value at ln(t) = 0 (t in hours).

## 3. Results

### 3.1. Microstructure

**Figure 1:**
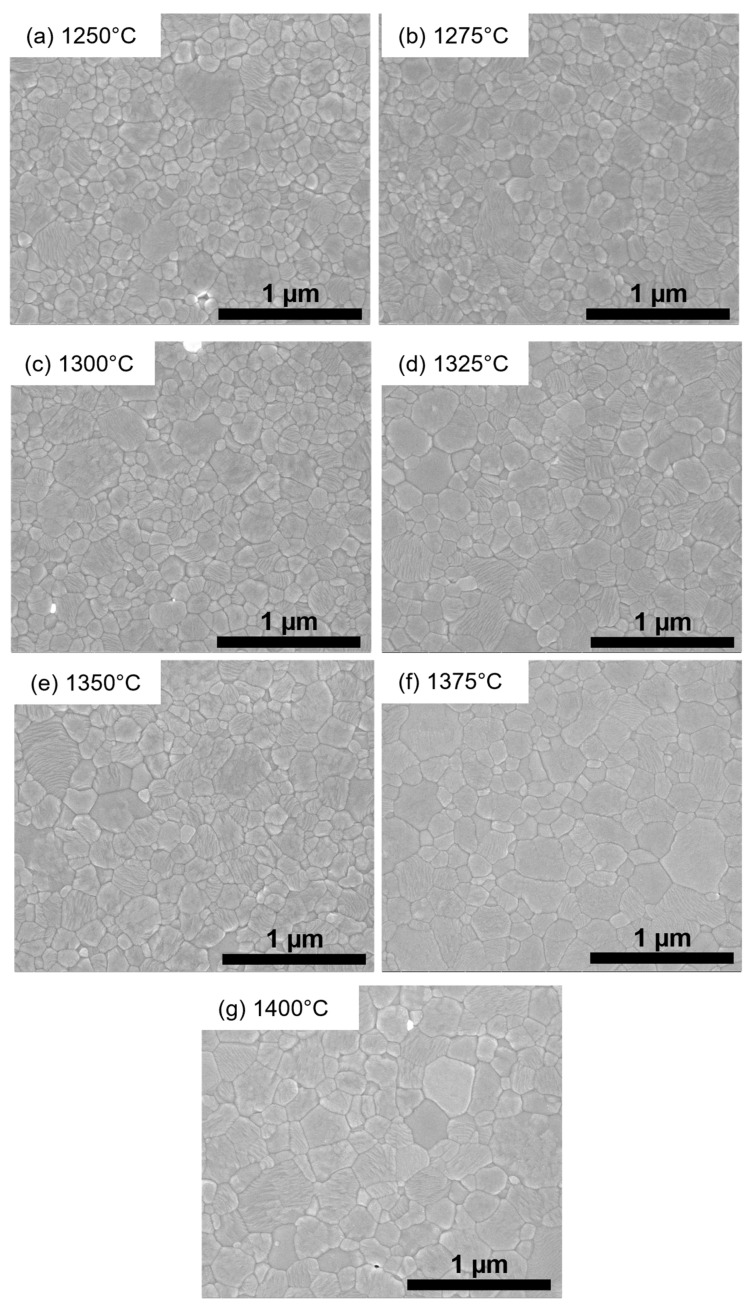
Microstructure of 1.5Y-2.2Ca-TZP materials sintered at different temperatures: SEM images of thermally etched surfaces. (**a**) 1250 °C; (**b**) 1275 °C; (**c**) 1300 °C; (**d**) 1325 °C; (**e**) 1350 °C; (**f**) 1375 °C; and (**g**) 1400 °C.

**Figure 2:**
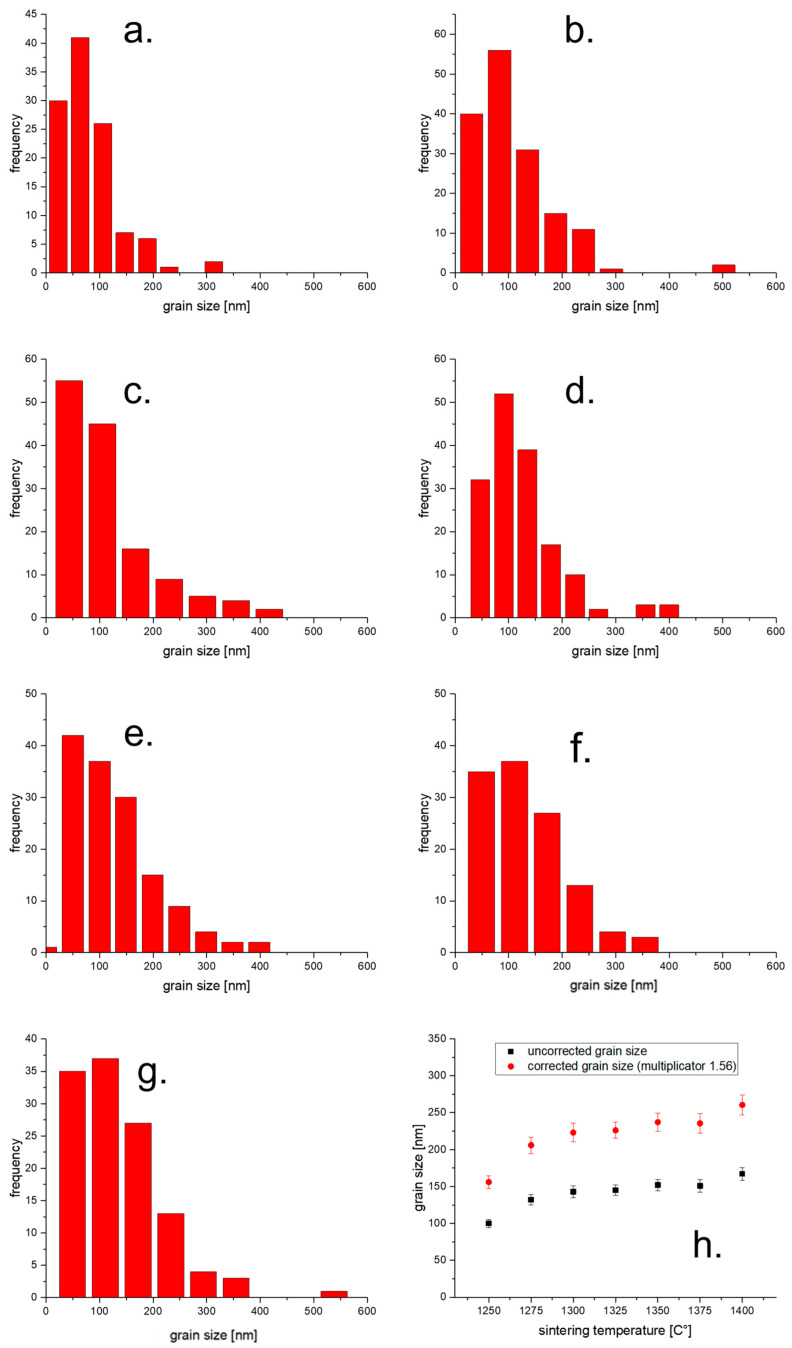
Histograms of grain size distributions (**a**–**g**). (**a**) 1250 °C; (**b**) 1275 °C; (**c**) 1300 °C; (**d**) 1325 °C; (**e**) 1350 °C; (**f**) 1375 °C; (**g**) 1400 °C. (**h**) Average grain sizes (uncorrected and corrected).

Figure 1 shows SEM images of polished and thermally etched surfaces of TZP sintered at 1250–1400 °C. Except for very few sporadic pores, the samples are dense and defect-free. A trend to coarsening with increasing sintering temperature is clearly visible. Figure 2a–g show histograms of the grain size distributions for the individual sintering temperatures. The average grain size (Figure 2h) increases from 150 nm at 1250 °C to 250 nm at 1400 °C. The grain growth in the range between 1275 and 1375 °C is very moderate.

### 3.2. Density and Mechanical Properties

Assuming a theoretical density of 6.03 g/cm^3^ (based on a density of 5.97 g/cm^3^ for 4.4Ca-TZP [30] and 6.08–6.1 g/cm^3^ for 3Y-TZP) the sintered samples showed a density of 99–99.6%. The maximum densities of 6.008 g/cm (99.6% TD) were measured at 1325–1350 °C. From the SEM images we may assume complete densification. The measured Young’s modulus values vary between 208.6 GPa and 211.9 GPa, and the measurement error is in the range between ±0.8 and 1 GPa. These values are typical for fully dense Y-TZP materials [32]. Poisson’s ratios are in the range between 0.321 and 0.323.

**Figure 3:**
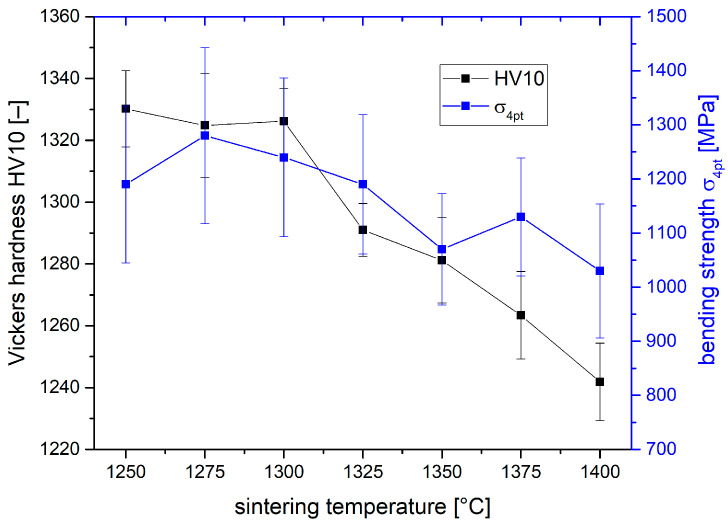
Hardness HV10 and 4 pt bending strength σ4 pt of 1.5Y-2.2Ca-TZP sintered at different temperatures.

Figure 3 shows the hardness and 4 pt bending strength of the TZP materials depending on sintering temperature. The Vickers hardness HV10 shows a continuous and steady decline with increasing sintering temperature from 1330 HV10 at 1250 °C to 1270 HV10 at 1400 °C. This is in good accordance with the trend of increasing grain size with rising sintering temperature. After an initial increase from 1180 MPa to 1280 MPa between 1250 and 1275 °C, the bending strength values subsequently decline to a value of 1050 MPa at 1400 °C.

**Figure 4:**
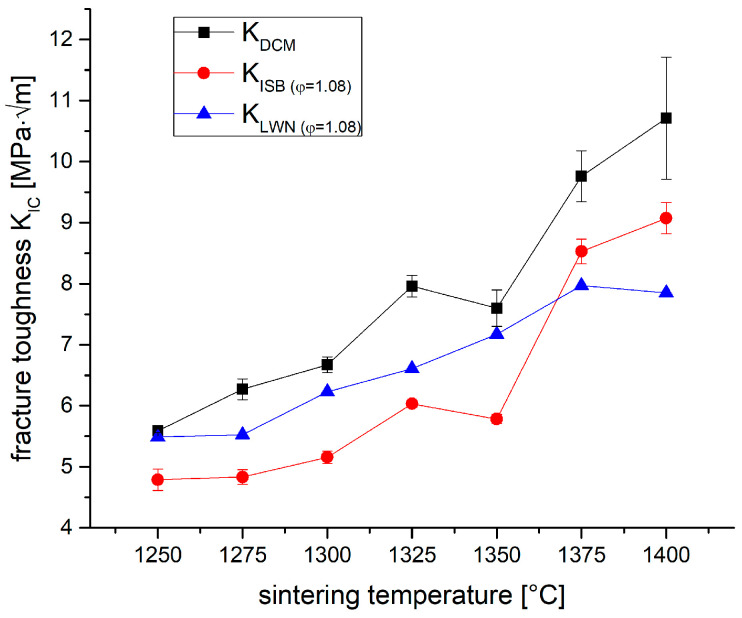
Fracture toughness K_IC_ of 1.5Y-2.2Ca-TZP sintered at different temperatures, determined by different methods.

Figure 4 shows the fracture toughness values determined by different indentation-based methods. The overall trend is identical for all methods. The toughness increases with increasing sintering temperature from a level of ~5 MPa√m at 1250 °C to 8–10 MPa√m at 1400 °C. The highest values were determined by direct crack length measurement. This method, however, bears the risk of crack trapping, especially for fine grain and very transformable materials [45]. The ISB and the LWN method are calculated based on residual strength. The LWN method also considers the length of extended cracks. Both ISB and LWN methods include crack geometry correction and appear therefore more reliable.

### 3.3. Phase Composition and Transformation Characteristics

**Figure 5:**
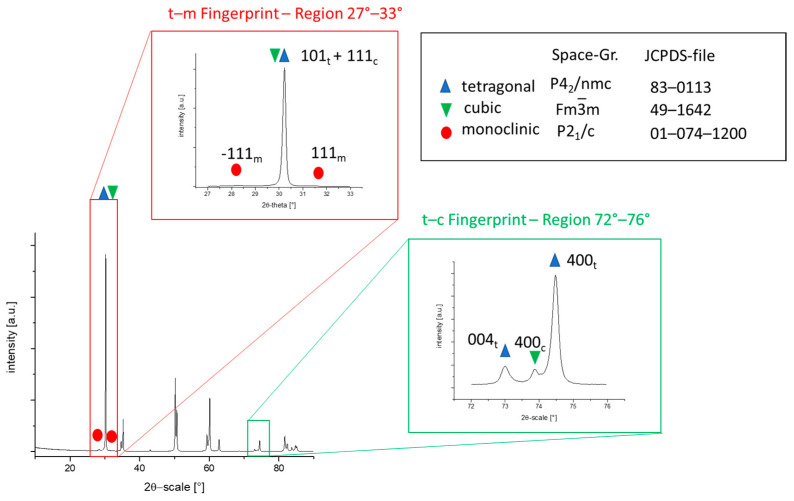
Full XRD-diffractogram of TZP sintered at 1325 °C with the magnified fingerprint regions studied in detail to quantify cubic and monoclinic fractions.

An example of a complete XRD diffractogram of TZP sintered at 1325 °C is given in Figure 5. The dominant structure is the tetragonal phase (PDF-card Nr.: 01-083-0113) [46]. Besides this tetragonal phase, a cubic phase (PDF-card Nr.: 00-049-1642) is present [47]. Due to the peak overlap at low 2θ-values, the tetragonal cubic ratio is determined in the t-c fingerprint region at 72–76°. Here, the cubic and tetragonal phases can be easily quantified. Small amounts of monoclinic phase (PDF-card Nr.: 01-074-1200) can be distinguished in the t-m fingerprint range at 27–33° [48].

**Figure 6:**
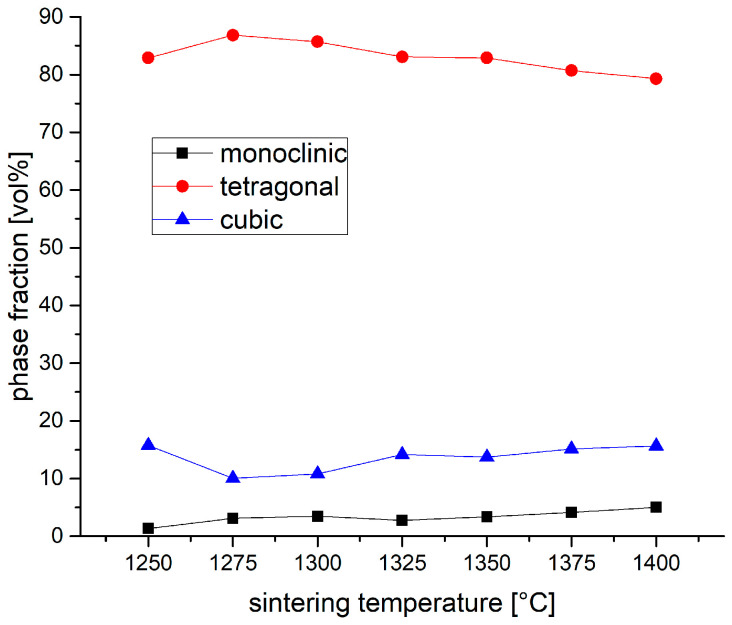
Phase composition (volume fractions) of polished 1.5Y-2.2Ca-TZP TZP samples sintered at different temperatures.

The complete XRD diffractograms of all samples tested covering the 2θ-range from 10 to 90° are provided in the Supplementary Material (Figure S1). The phase composition determined on polished TZP samples sintered at different temperatures is shown in Figure 6. The monoclinic content rises from 1.3 vol.% to 5 vol.% between 1250 °C and 1400 °C. This may—at least partly—originate from the sample preparation process. Lapping and even gentle polishing processes may lead to a slight phase transformation in the machined surface. The cubic content decreases from 16 vol.% to 10 vol.% between 1250 °C and 1275 °C sintering temperatures. Then, the cubic content rises steadily to 16 vol.% at 1400 °C. We may assume that the high initial cubic content at 1250 °C could be an artifact of the explosion synthesis process of the 3Y-TZP [34]. The progressive increase in cubic phase is in good accordance with the rising influence of thermodynamics. Higher sintering temperatures favor the segregation of the cubic phase. Accordingly, the tetragonal phase fraction has a maximum of 86 vol.% at 1275 °C and declines towards higher sintering temperatures to 79 vol.% at 1400 °C.

**Figure 7:**
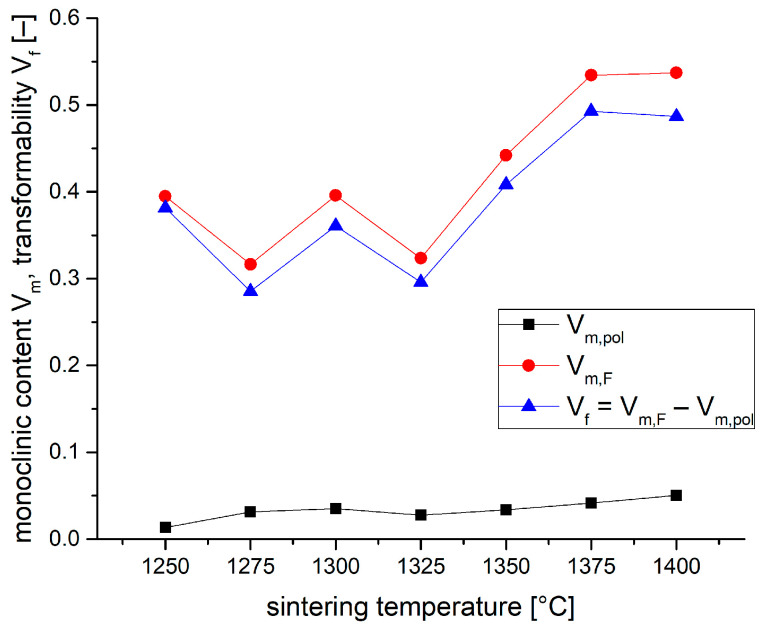
Monoclinic contents in polished (V_m,pol_) and fractured surfaces (V_m,FF_) of 1.5Y-2.2Ca-TZP samples sintered at different temperatures.

Figure 7 plots the monoclinic contents in polished V_m,pol_ and fractured surfaces V_m,F_. The difference between both values is the transformability V_f_. V_f_ increases from values of 30–35 vol.% at sintering temperatures of 1250–1325 °C to a value of 50 vol.% at 1375–1400 °C. The higher fluctuations in the values of samples sintered at low temperature are related to the relatively rough and uneven fracture surfaces of these samples, which have a high strength and moderate toughness. They show catastrophic failure and frequently exhibit multiple fractures. The tougher samples sintered at 1350 °C and higher did not show multiple fractures. Their fracture surfaces were considerably smoother, which provides more reliable XRD results.

**Figure 8:**
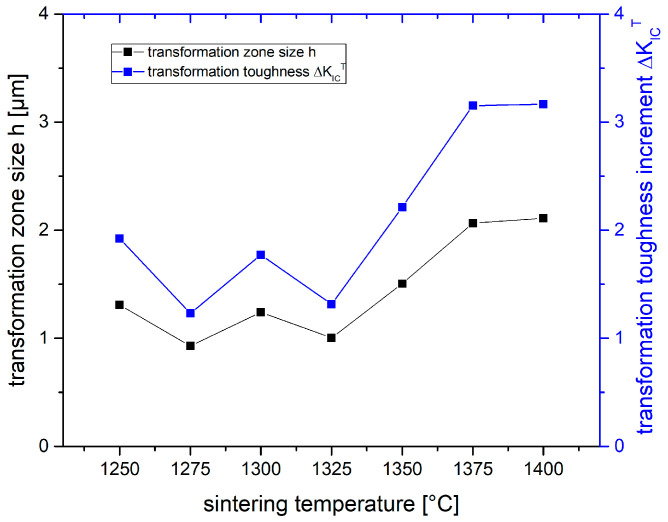
Calculated transformation zone sizes h and the transformation toughness increments ΔK_IC_^T^ of 1.5Y-2.2Ca-TZP samples sintered at different temperatures.

Figure 8 shows the transformation zone sizes h and the transformation toughness increments ΔK_IC_^T^ calculated from the above XRD results. Note that the above-described fluctuations in monoclinic contents of fractured surfaces are transferred to values of h and ΔK_IC_^T^.

The samples sintered at low temperatures (1250–1325 °C) show only moderate transformation zone sizes of 1–1.2 µm coupled with a moderate transformability. Hence, a low transformation toughness increment ΔK_IC_^T^ of 1.2–1.8 MPa√m is calculated. The samples sintered at 1375 °C and 1400 °C show zone sizes of 2 µm and transformation toughness increments of 3.2 MPa√m. Assuming an intrinsic toughness K_0_ of 4 MPa√m [16] and no other toughening effects except transformation toughening, we may assume that K_IC_ = K_0_ + ΔK_IC_^T^ [49]. The toughness values calculated from XRD data are in the range between 5.3 and 7.2 MPa√m and follow the same rising trend with sintering temperatures as the measured toughness values shown in Figure 4. The best match between measured and calculated toughness values is observed for K_LWN_.

### 3.4. Low-Temperature Degradation Resistance

**Figure 9:**
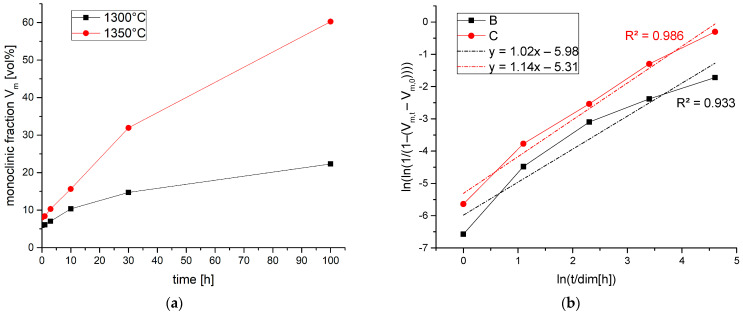
Low-temperature degradation behavior in an accelerated autoclave test at 134 °C. (**a**) Monoclinic fractions Vm of samples sintered at 1300 °C and 1350 °C at different aging times in autoclave test at 134 °C, and (**b**) MAJ-plot of data shown in Figure 9a.

The monoclinic contents of samples sintered at 1300 °C and 1350 °C after different treatment times in an autoclave at 134 °C in saturated water vapor are shown in Figure 9a. The trends do not show the typical sigmoidal trend [44]. After an initial steep rise, the monoclinic content tends to saturate. The LTD resistance of the material sintered at 1300 °C is considerably higher; here, the monoclinic content after 100 h is only 20 vol.%. The sample was still intact. The sample sintered at 1350 °C showed a monoclinic content of >60 vol.% at 100 h and was broken. Figure 9b shows an MAJ plot of the data [50]. A linear regression over the whole range to determine the nucleation factor is not possible. The slope decreases with increasing aging time, which indicates a mechanistic change. The results show a strong similarity to the results obtained with explosion-synthesized 3Y-TZP [32], which also showed a mechanistic change after 10 h of autoclave aging. The comparison of the present data with pre-existing results shows an LTD stability between Ca-TZP (no aging) [29] and detonation-synthesized 3Y-TZP [44]. Interestingly, the nucleation factor determined in this study, which is approximately n = 1, was also found for the detonation-synthesized 3Y-TZP. N = 1 means zero-order growth, i.e., the layer thickness increases with a constant speed. This contradicts the typical nucleation and growth scenario depicted for Y-TZP (with n = 4). The result is, however, in good accordance with results published by Keuper et al. [14]. The rate constant lnb, however, was lnb = −4 for 3Y-TZP [44] and was between b = −5.3 and b = −6 in the current study. This means the Y-Ca-TZP sintered at 1300 °C ages by a factor of ~7 slower than the 3Y-TZP sintered at 1400 °C. The fine grain size of the YCa-TZP is probably one of the main reasons for the enhanced LTD resistance. According to Chevalier’s interpretation [51], the LTD process is initiated by the transformation of isolated grains at the surface. The volume expansion of this first grain weakens the grain boundaries of the surrounding grains. Hence, the penetration of the water into the bulk is facilitated and causes the growth of the transformed regions into the bulk. As the transformed region expands, the stress on the surrounding grains constantly increases, which leads to a high Avrami exponent n. In the case of small grains (here: 200–220 nm, standard Y-TZP: 400–500 nm), the initial stress exerted by the first transformed grain is much lower (the stresses scale with R^3^) and the growth of the transformed region is thereby impeded. Therefore, an ultrafine initial grain size is favorable for high LTD resistance. The different grain boundary chemistry with a mixture of yttria and calcia at the grain boundary may also be of importance. Ca^2+^ (114 pm) has a much higher ionic radius than Y^3+^ (104 pm) and consequently tends to segregate more to the grain boundary. Stabilizer supersaturation at the grain boundary is known to be beneficial to LTD resistance.

## 4. Discussion

The results from the study performed by mixing and milling Ca-TZP and Y-TZP to obtain a co-stabilized Ca-Y-TZP material show that the concept can be successfully applied. This simple procedure facilitates the manufacturing of Y-Ca-co-stabilized TZP materials using commercially available Y-TZP and Ca-TZP powders as raw materials. However, the range of possible compositions is somewhat limited, as presently, only one Ca-TZP powder is available, and because the available Y-TZPs are mostly 2Y-TZP and 3Y-TZP powders.

The mechanical properties and aging resistance of the mixed material range between the properties of 3Y-TZP and 4.4Ca-TZP. At low sintering temperature, the materials are strong and relatively brittle but very LTD-resistant. At higher sintering temperature, the toughness increases notably to a very attractive level above 7 MPa√m—at the expense of lower strength and LTD stability, however.

The phase analysis shows that the enhanced toughness at high sintering temperatures is a result of a higher transformability. This higher transformability can be traced back to the increase in grain size but also to the segregation of the cubic phase. The formation of the cubic phase leads to a stabilizer depletion of the tetragonal phase and favors higher toughness. However, this also seems to be the clue to the reduced LTD resistance at higher sintering temperature.

The analysis of microstructure shows a progressive elimination of ultrafine grains at increasing sintering temperature. The microstructure, however, remains extremely fine-grained. The grain sizes are significantly lower than in 3Y-TZP sintered under identical conditions [29] but coarser than in 4.4Ca-TZP [30]. Co-stabilization with calcia seems to efficiently suppress grain growth by solute drag. As we may expect, the initial composition of separate Ca-TZP and Y-TZP grains does not equilibrate instantaneously. This is, however, the only apparent disadvantage in comparison to Y-Ca-TZP made from a single co-stabilized precursor.

Based on this first basic experiment, we think that it will be worth trying different Y-Ca-TZP compositions by variation in the yttria-stabilized zirconia starting powders (different BET-surface, different stabilizer content) and their fraction. This will lead to a deeper understanding of the relations between composition, microstructure, phase composition and mechanical properties of this new co-stabilized TZP system. Eventually, if the 4.4Ca-TZP should become an established standard material, other Ca-TZP powders with different stabilizer contents may become available. This would broaden the parameter space for elaboration of Y-Ca-co-stabilized TZPs considerably.

## 5. Conclusions

Yttria-calcia-co-stabilized TZP materials combine excellent mechanical properties with high LTD resistance. The mixing and milling technology of commercially available starting powders provides easy access to standard compositions. The extreme grain microstructure with a relatively broad grain size distribution at low sintering temperature indicates an initially inhomogeneous distribution of stabilizers and a progressive equilibration toward higher sintering temperatures. The strength and LTD resistance show an inverse correlation with fracture toughness. Low sintering temperatures favor strength and LTD resistance, while high sintering temperatures provide high fracture toughness. The LTD and mechanical properties of co-stabilized material lie in-between the properties of 3Y-TZP and 4.4Ca-TZP. Y-Ca-TZP materials may be interesting alternatives to Y-TZP in biomedical applications such as dental implants.

## Data Availability

The original contributions presented in this study are included in the article/Supplementary Material. Further inquiries can be directed to the corresponding author.

## Supplementary Materials

The following supporting information can be downloaded at https://www.mdpi.com/article/10.3390/ma19061205/s1, Figure S1: XRD diffractograms of polished samples in the 10–90° 2θ-scale.

## Abbreviations

The following abbreviations are used in this manuscript:

TZP	Tetragonal zirconia polycrystals
SEM	Scanning electron microscopy
MAJ	Mehl–Avrami–Johnson
XRD	X-ray diffraction
LTD	Low-temperature degradation
