# Supplementary material for: Yttria-Calcia-Co-Stabilized Tetragonal Zirconia Polycrystals Made by Powder Mixing
Source: Materials (Basel). 2026 Mar 19;19(6):1205. doi: 10.3390/ma19061205 (PMC13027816; doi:10.3390/ma19061205)
Supplement: Supplementary file 1 [file materials-19-01205-s001.zip › materials-4162894-supplementary.pdf]

Supplement to: Yttria-calcia-co-stabilized tetragonal zirconia polycrystals made by powder mixing

Selina Grübel [1], Bettina Osswald [1] and Frank Kern [1*]

XRD data:

Figure S1 shows the XRD diffractograms of polished surfaces of YCa-TZP materials sintered at 1250°C -1400°C (2θ-range 10°-90°).

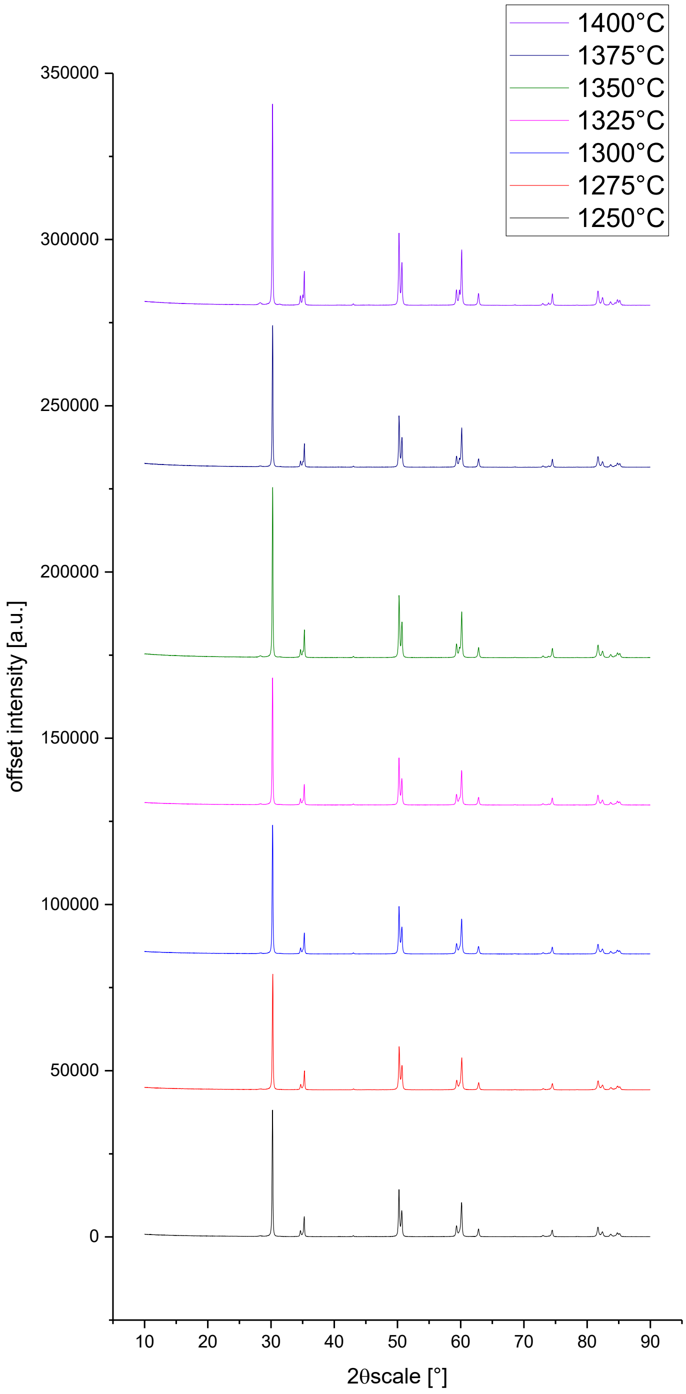

Figure S1. XRD diffractograms of polished samples in the 10-90° 2θ-scale range
